# Losing independence – the lived experience of being long-term sick-listed

**DOI:** 10.1186/1471-2458-13-745

**Published:** 2013-08-12

**Authors:** Linda Lännerström, Thorne Wallman, Inger K Holmström

**Affiliations:** 1 Department of Public Health and Caring Sciences, Family Medicine and Preventive Medicine Section, Uppsala University, Uppsala, Sweden; 2 Centre for Clinical Research Sörmland, Uppsala University, Eskilstuna, Sweden; 3 School of Health and Medical Sciences, Örebro University, Örebro, Sweden; 4 Department of Public Health and Caring Sciences, Health Services Research Section, Uppsala University, Uppsala, Sweden

## Abstract

**Background:**

Sickness absence is a multifaceted problem. Much is known about risk factors for being long-term sick-listed, but there is still little known about the various aftermaths and experiences of it. The aim of this qualitative study was to describe, analyze and understand long-term sickness-absent people’s experiences of being sick-listed.

**Methods:**

The design was descriptive and had a phenomenological approach. Sixteen long-term sickness-absent individuals were purposively sampled from three municipalities in Sweden in 2011, and data were collected through semi-structured, individual interviews. The interview questions addressed how the participants experienced being sick-listed and how the sick-listing affected their lives. Transcribed interviews were analysed using Giorgi's phenomenological method.

**Results:**

The interviews revealed that the participants’ experiences of being sick-listed was that they lost their independence in the process of stepping out of working society, attending the mandatory steps in the rehabilitation chain and having numerous encounters with professionals. The participants described that their life-worlds were radically changed when they became sick-listed. Their experiences of their changing life-worlds were mostly highly negative, but there were also a few positive experiences. The most conspicuous findings were the fact that stopping working brought with it so many changes, the participants’ feelings of powerlessness in the process, and their experiences of offensive treatment by and/or encounters with professionals.

**Conclusions:**

Sick-listed persons experienced the process of being on long-term sickness absent as very negative. The negative experiences are linked to consequences of stopping to work, consequences of social insurance rules and to negative encounters with professionals handling the sickness absence. The positive experiences of being sick-listed were few in the present study. There is a need to further examine the extent of these negative experiences are and how they affect sick-listed people’s recovery and return to work. Long-term sickness absence; sick leave; experiences; interviews; phenomenology; Sweden.

## Background

Some people are so hard struck by illness that they cannot continue working. They become sick-listed, and hence begin being absent from work. In Sweden 4.6% of those who work are long-term sickness absent [1]. Inability to work due to illness and the subsequent economic loss cause a great deal of problems in Western welfare societies. For the past decade, the Swedish state has struggled to decrease one of Europe’s highest sickness absence rates by introducing alterations to the rules for sickness benefits as well as working with quality and efficiency in both the management and rehabilitation of people with illness. Sickness absence is a multifaceted problem. Much is known about risk factors for being long-term sick-listed, but there is still too little known about its various aftermaths [2,3].

In Sweden, a sick person needs a physician’s certificate to be put on sick leave upon the eighth day of sickness. To issue a sick-leave certificate, the physician has to diagnose the person and determine his or her capacity to work. The employer finances the sickness benefit for the first two weeks, and after this the state – the Swedish Social Insurance Agency – takes over the responsibility and continuously assesses whether the sick person is entitled to sickness benefits. This assessment is based on whether the person’s work ability is impaired due to disease and is, for the first six months, related to his or her current workplace. After this time, the work ability determination is related to the entire labour market [4].

Previous research describes that people who are sickness absent face changes in areas like health, work, relations with family and friends, and economy [2,3,5-7]. Ockander and Timpka [8,9] interviewed 82 middle-aged, long-term sick-listed (>60 days) women and the aim was to explain how the absence arises and becomes permanent. They describe that in the beginning of the absence the experience was positive and a chance to rest. After some time, however, the experience turns from positive into a negative circle of pain, inactivity and isolation, which leads to indifference. Other studies describe that the period of sickness absence changes a person’s self-image and life goal/life rhythm, and causes feelings of exclusion, shame and social stigma [6,7,10-16]. Fifteen men and women were interviewed by Hansen Falkdal et al. [11] exploring what was experienced as important in their process of returning to work, moving to long-term sick-leave or receiving a disability pension. Predictors for returning to work or disability pension were: mental resources, clear or unclear diagnosis, time spent in sick-leave process and personal belief in ability to work. Edwards and Gabbay [10] interviewed 26 men and women and the findings agree that motivation to work and personal circumstances is important for return to work. In focus group discussions with 16 men and women Jansson and Björklund [12] explored returning to work from an environmental perspective. They concluded that difficulties in returning to work could not be reduced to individual factors but as an interactive problem involving individual, structural and environmental aspects.

After a few months of sickness absence, the probability of returning to work decreases drastically [2,6,17]. The rapid deterioration of the probability of returning to work indicates that sickness absence – and particularly long-term sickness absence – entails processes that somehow undermine and decrease people’s work ability and health and cause all the above-mentioned changes; research on people receiving disability pension confirms this. Disability pension receivers have higher health care utilization, lower quality of life and higher risk of premature mortality than do people who work, even when the disease causing the disability is adjusted for [13]. There is also evidence that work is generally good for one’s general well-being, mental and physical health, and that unemployment is strongly associated with poorer health [18].

Although sickness absence is a large problem, little research has engaged in describing the experiences of the sickness-absent people and the accompanying changes in life. Through studying experiences, an approach often used within life-world research for studying illness [19-23], keys can be found which clarify and give incentives for interventions at critical points of the sick-listing process. The aim of this qualitative study was to describe, analyze and understand long-term sickness-absent people’s experiences of being sick-listed.

## Methods

The study used a qualitative and descriptive design with a phenomenological approach. Phenomenology is a qualitative method which arose from the philosophical work of Husserl [24]. The method offers a way to gain knowledge of people’s experiences of different *phenomena*. Experiences of phenomena take place in people’s everyday life in their *life-world*; that is, people’s subjective, perceivable and unreflected world in which they live. Phenomena can be explored through studying people’s meaning-making of them in their life-world. Meaning is created in processes in a person’s consciousness; these processes are always directed (*intentionality*) at something (*a phenomenon*). In the study of phenomena, it is important to go back “to the things themselves” [24] and describe them as they are. By adopting a *phenomenological attitude* and thus *bracketing* (*reducing*) preconceived assumptions, theories and thoughts, the researcher can do better justice to the phenomena and describe them as they are experienced. In the scientific description, when reflecting on the people’s meaning-making experiences of a phenomenon, an essential meaning structure and sometimes a core, *an essence*, of the phenomenon can be revealed and described [25-28]. The phenomenon in this study was sick-listing. What does it mean to be sick-listed? What does it mean to not be able to work because of illness? What are the aftermaths of life as a sick-listed person? How is one’s life-world changed?

### Sample

The study was conducted in three municipalities in central Sweden in 2011. The sampling was purposive with the goal of maximum variation to attain diverse lived experiences and perspectives of long-term sick listing. The municipalities were chosen based on their differing levels of sickness absence: one is rated low, one medium and one high. All individuals, 37 persons, at three public health care centres, sick-listed for more than three months were invited to participate. Initially 17 long-term sick-listed individuals agreed to participate. One of the participants who agreed to take part did not appear at the interview place and then declined. Twenty of the invited declined due to unknown reasons (N=16) or did not answer the phone (N=4). Nine of the decliners seemed to be of foreign origin (based on their names). The final sample comprised of 16 participants, nine women and seven men; a little more than 40 percent of those invited (Table 1). Nine of them were fully sick-listed, while seven were part-time sick-listed. They were aged between 31 and 64 years, and had varied educational levels. Three had been born abroad, but had lived in Sweden since childhood. Before being sick-listed eleven of the participants were employed, three had their own company, one was a student, and one was unemployed. The self-reported causes of the sick-listing were back problems, psychiatric problems and cardiovascular problems.

**Table 1:** Demographics of participants Cause of sick leave, self-reported: 1 Back/neck; 2 Depression; 3 Other mental diagnosis; 4 Cardiovascular; 5 Accident; 6 Other Education, self-reported: 1 Elementary school or equivalent; 2 2 years of high school or vocational school; 3 3–4 years of high school; 4 University or college, 2.5 years or shorter (<180 HP); 5 University or college, 3 years or longer (≥180 HP); 6 Other education

**No**	**Gender**	**Age**	**Months of sick leave**	**Degree of sick leave**	**Cause of sick leave**	**Ethnicity**	**Education**
1	Male	43	5-11	Part-time	1, 5	Foreign	2
2	Male	61	3-5	Part-time	4, 5	Foreign	1
3	Female	62	>12	Part-time	1, 2, 3	Swedish	3
4	Female	57	>12	Part-time	1, 5	Swedish	3
5	Male	60	3-5	Full	1, 5, 6	Swedish	2
6	Female	37	>12	Full	3	Swedish	5
7	Female	48	>12	Part-time	1, 2, 3	Swedish	2
8	Male	53	>12	Part-time	2	Swedish	6
9	Female	63	>12	Full	4	Swedish	4
10	Male	61	>12	Full	6	Swedish	1
11	Female	36	>12	Part-time	2	Swedish	3
12	Female	63	5-11	Full	6	Swedish	5
13	Male	46	5-11	Part-time	1	Swedish	2
14	Male	52	>12	Full	4	Swedish	2
15	Female	64	>12	Full	6	Foreign	5
16	Female	31	>12	Part-time	1	Swedish	1

### Data collection

The data were collected through semi-structured individual interviews to gain rich descriptions [29]. The participants were contacted by email and invited to attend an interview. In the letter the participants were informed that participation was voluntary and were told of the measures that would be taken to protect their identity. After two weeks the participants were contacted via telephone so that they could consent or decline participation. The interviews were performed at a learning centre and at two libraries near the participant’s residence. At the interviews, the participants were orally informed of the above and were also asked to sign informed consent. They were also informed about the aim of the study. Further, they were asked to complete a form with demographic questions. The average duration of the interviews was 40 minutes (range 16–61), and they were recorded digitally. The first question in the interview was “Can you tell me how it was when you became sick-listed?”, and was followed by questions concerning how the sick-listing had affected their life, asking them to describe experiences in the process (Table 2). The study was approved by the Local Committee for Research Ethics in Uppsala, DNr 2011/131-31/5.

**Table 2:** Interview guide

	
1	Can you tell me how it was when you became sick-listed? How did it feel?
2	Has the sick-listing changed your life? In what way?
3	Has it changed the way you look at yourself?
4	Where there something that could have been done to prevent you from being sick-listed?
5	How do you picture your future?
6	Do you feel you have had an influence on your own sick-listing?
7	How did you feel when you met the physician; physiotherapist; behaviourist; Social Insurance Officer; employer/Public Employment Officer
8	Have you participated in a reconciliation meeting? Can you describe it? Did you feel that you could influence the decisions made?

### Data analysis

The interviews were verbally transcribed and analysed using Giorgi’s phenomenological method [25-28]. The analysis followed the four steps of Giorgi’s analysis process:

1. **Reading the data:** All the printed interviews were read several times by all authors (two nurses and one physician) until a sense of the whole was attained.

2. **Breaking the data into parts:** The text was divided into meaning units by the first author, discriminating units that highlighted the experience of being sick-listed. In this step, the analyser tried to embrace the phenomenological attitude and bracket the preconceived perceptions of how it is to be sick-listed. The analyser tried to be what Giorgi describes as “discovery-oriented” and sensitive to including unexpected descriptions. The question “Does this describe how it is to be sick-listed?” was used as help in this step. The descriptions were put into a scheme.

3. **Organizing the data:** All the meaning units were examined, probed and re-described in a more scientific language while still revealing the original experience. The descriptions formed “revelatory themes”. The question “What does it mean to be long-term sick-listed?” was used to remain focused on the aim of the study. Many of the themes coincided and could be merged, and it appeared that they belonged to three different main themes.

4. **Expressing the structure of the phenomenon:** The essential revelatory themes, along with the main themes, were laid out for an overall scrutinization, after which an essential structure of the phenomenon emerged (Table 3). To find the essence, the main themes were used as questions, for example “What does it mean to step out of working society? What does it mean to follow the steps in the rehabilitation chain and to have numerous encounters with professionals?”

**Table 3:** Analysis structure

**Essence**	**Main theme**	**Revelatory theme**
**Losing independence** Independence was lost when the participants, due to illness, had to stop working and start relying on the state for support. The uncertain waiting to heal, the absence from work and the conditions for support from the state caused processes that for most of the participants impaired their confidence in themselves and authorities.	Stepping out of working society	Rest
		Waiting in uncertainty
		Changed self-perception
		Stigmatization
		Changed economic conditions
	Following the steps in the rehabilitation chain	Feelings of powerlessness
	Numerous encounters with professionals	Being questioned
		Getting mixed messages and sitting in-between
		Disrespectful encounters
		Respectful encounters

## Results

**Figure 1:**
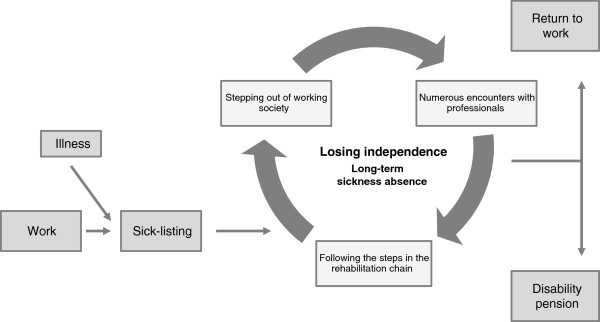
The location of the findings in the sickness absence process.

The essential meaning of being sick-listed was the loss of independence (Figure 1). Independence was lost when the participants, due to illness, had to stop working and start relying on the state for support. Most of the participants experienced their present life as a constant, uncertain “pending” while being questioned by authorities and society. They were all exposed to the social insurance rules and obliged to follow the steps in the rehabilitation chain but it affected them differently. A few experienced being treated well in encounters with professionals and went through changes that ultimately led to a better life, whereas many experienced not being treated well, which caused impaired confidence in themselves and in authorities.

### Stepping out of working society

The participants in this study experienced that their illness pushed them to the point where they could no longer cope with the effort required to work. They had to stop working to start healing; even though they wanted to work, they were not able to.

#### Rest

The sick-listing turned into relief, when they finally got the possibility to rest after having struggled for a long time. They described rest as a prerequisite for gaining energy to cope with living and start healing. Some described being unaware of the need to rest but being urged by relatives to seek care.

#### Waiting in uncertainty

Several of the participants could not recall how they had spent their first months of being sick-listed. Their memories of this time were unclear and blurred. After the first period, the waiting started – it was long, and it was a waiting in uncertainty. Some described knowing what they were waiting for, for instance to see a specialist or to undergo a neuropsychiatric examination, while others did not know. Some were waiting for their body to heal. The uncertainty was described as stressful, and as causing worry that they would not know when, or if, their body would regain its former strength or if they would get an appointment with the specialist. The waiting filled their days, and they described that after a time without routines they became passive, inactive, unmotivated and even apathetic. The experience of lacking the power of initiative was especially unpleasant for those who were used to be very driven and energetic, and it caused them to question their own abilities. Many of the participants experienced reversing or displacing day and night. This resulted in their sleeping either too much or too little, which contributed to inactiveness. Some of the participants were part-time sick-listed and could continue working. They thus still had contact with the work and routines but also more time to rest, which was described as positive. The part-time sick-listing gave them the opportunity to gain energy to live better. The prolongation of the sick-listing certificate was a cause of worry. Often, it was prolonged only for a few weeks or months, and every time it needed to be prolonged there was a worry about whether or not it actually would be. Several of the participants described feeling pressured to heal more quickly:

I8: I have nothing to complain about; I’ve been extremely lucky with officers and doctors and so on… I’m totally vulnerable, it can turn at any moment, I have no control whatsoever.

L: Control?

I8: Well this as you say is a right…it…for me it doesn’t exist…it’s like some kind of grace period all the time.

L: Mmm

I8: And it’s the same trauma when…ehh…when a sick-listing spell ends and it’s time to see the doctor and that…I don’t know beforehand if it’s going to work or…it’s…like playing the lottery. (Interview 8)

#### Changed self-perception

All of the participants expressed that they wanted to work, but that their body and the illness symptoms restrained them*.* The feeling of wanting to work but not being able to was described as not being a complete human being or as being a second-class human being. The things they used to cope with, and used to do well, no longer worked. This changed their perception of themselves. A woman expressed this by saying:

I12: It’s just that it feels weird…to…not be sound so to say…

L: Mmm. What do you mean by sound?

I12: Work full-time (pause)

L: And when it feels weird…what…how does it…how does it affect you?

I12: That I have to rethink things

L: Mmm in what way?

I12: ”The little girl who could” doesn’t exist anymore…….and maybe that’s good…it’s probably…it’s probably a development (Interview 12)

The process from being able to work and provide for oneself to becoming a person unable to do this was described as frustrating. The participants described work as very positive and important for their self-confidence and self-esteem. It was important because they got to see others and interact socially, which gave them a great deal of satisfaction and strengthened their self. Work also boosted their self-esteem because they could deal with the tasks they were assigned there. Having tasks they needed to do made them feel needed and worthy.

#### Stigmatization

Being sick-listed was accompanied by a feeling of being questioned by both society and the authorities. Some participants described that society started to look at them differently when they became sick-listed. They expressed being stigmatized and expected to behave and look in a certain way. Some participants heard rumours of people questioning the severity of their illness and saying they looked too healthy to be ill. They believed it would have been easier to have a condition that could be seen, like a broken leg. Some had heard people imply that they were sick-listed so that they could take care of their children. Other participants described embracing unspoken rules to not go out and work in the garden, or even go out at all, in fear of being seen as too healthy.

I7:…so sure, you felt this stamp on you…you might not want to go out in the garden…or be seen out at all…you’ve been standing behind the curtain and looking, no one should be able to see…it would be better if you’d broken an arm, then it shows…and then it’s boring to go around like this…it’s horrible…it’s terrible.

L: Tell me what’s boring?

I7: Well everybody else goes to work…the children go to school…there I am…although I have…I’m not a person who sits around, so I do…I do things so I’ve had things to do anyway, I mean I have four children and all this so I’m not going to try to hide that…but anyway…then I started to feel that I want to have colleagues, I want to get out too … I want to have social contacts too, I want to be able to be out in my garden without anyone seeing me or making an ugly face…’now she’s out in the garden’ and all that…

L: Have you experienced that?

I7: Yes, yes, yes. I’ve hidden the whole time…nobody should be able to see…

L: What do they say?

I7: Well…she’s at home to take care of her children…ah…it doesn’t show…oh no, there have been many mean tongues I have to say, so it’s been very tough periodically.

(Interview 7)

#### Changed economic conditions

Most of the participants’ economic conditions had changed for the worse. With the reduction in income came changes in life. Some could not continue eating the same food they used to, but instead had to chase discounts and eat cheaper food. Many had to give up buying clothes and entertainment. One participant described that she had used all her savings to pay the bills; this was money she had saved for herself over a 20-year period. One participant described keeping the house at only 15 degrees Celsius during the winter to save on the heating costs. Several feared that they would need to move to a cheaper home if the sick-listing continued. The deterioration in economy was described as a failure, as they could no longer provide for their family. The feeling of not being able to support their family was hard to bear, and made them feel worthless and ashamed:

L: Do you think it’s affected the way you look at yourself?

I1: It’s affected everything.

L: How do you look at yourself compared to before?

I1: You’re a loser.

L: It feels like that?

I1: Yes, of course you are.

L: For not being able to work?

I1: You can’t work, you can’t make money, everything, you can’t get help. (Interview 1)

Although most of the participants received noticeably less money, there were a few examples of participants who stated that they had no financial problems whatsoever as a result of the sick-listing. One participant even had better economic conditions thanks to private sickness insurance.

### Following the steps in the rehabilitation chain

Being sick-listed meant being obliged to follow the steps in the rehabilitation chain to be entitled to support and rehabilitation. A woman described how she experienced this:

…it feels like when you get sick-listed you’re drawn into a system that you yourself can’t influence, it’s just some kind of…something you have to, have to go through…I can’t do anything to influence the order of how it works; it’s others who have all the power over me. (Interview 6).

#### Feelings of powerlessness

Most of the participants described feelings of powerlessness in the process of being sick-listed. They felt forced to follow the steps in the chain, as they would not get their allowance otherwise. For some the steps included rehabilitation measures, while others passed through the system with no rehabilitation at all. Some wanted rehabilitation but did not get it. They felt they had to actuate and sometimes perform their own rehabilitation. Others were offered rehabilitation but did not want it, and felt forced to participate. One participant described not wanting to have acupuncture because she had a fear of needles, and consequently declining. She was then accused by her physician of not wanting to get well. She described feeling very offended at this.

The feeling of powerlessness also concerned getting a new job if the old one was unsuitable. If in need of a new job, participants were assigned one by the Public Employment Service. Several participants said it did not matter what they wanted or thought. One participant described being very pessimistic about participating in a labour market measure, but was positively surprised and got a job as a result of this. Some felt offended by work suggestions which could, for example, require a much lower level of competence than they were used to or be in an area they were not interested in.

I10: They think I should go to the moron factory as I understand it and sit down and count nails in a bag…ehhh…it doesn’t feel like they care more than saying ’No, now he has to go and work’. And what I’m going to work with they haven’t said; they’re going to try together with the Employment Service…to…get something going…as I don’t feel…as I don’t feel it’s worthwhile in my brain anyway…so there we are…it’s almost a degrading suggestion. I’m still not stupid.

L: Mmm. But how do they respond when you say that?

I10: I don’t think they understand, they say I’m not the one who decides but it’s rather the Employment Service and Social Insurance Agency who decide, and this coach I have who I still haven’t met…a work coach for a man who’s been self-employed since 1975 and has been in 15 different industries, it feels like – Okay, I said, you can take care of the paperwork cause I can’t…but the physical part in the field – that I can do. They haven’t accepted that as I understand it…(Interview 10)

### Numerous encounters with professionals

Being long-term sick-listed entailed being exposed to numerous encounters with professionals – these could be physicians, rehabilitation professionals, officers at the Social Insurance Agency, and sometimes officers at the municipality and the Public Employment Service.

#### Being questioned

The professionals described above were assigned to question the sick-listed individual based on their authority’s viewpoint and assignment, in relation to the sickness insurance during the sick-listing process and rehabilitation. Being continuously questioned caused most of the participants to feel they were not believed or listened to, and as if their credibility was questioned. A woman described a reconciliation meeting (meeting with the sick-listed person, the Swedish Social Insurance Agency, the employer or the Swedish Public Employment Service, the physician and sometimes the municipality to plan rehabilitation):

Because you feel…well if you feel nervous before such a meeting you feel totally flattened afterwards. And then it’s the jargon, especially from the Insurance Agency…they…I usually say…how does it feel people ask me…I feel like I’m a registration number…(pause)…that’s how it is. I feel that I’m…yes…I’m a number. I feel like a prisoner. And they have their norms…ehh…after this amount of time you’re supposed to be here and after that amount of time you’re supposed to be there…and maybe you can be that if you’ve broken an arm…there are no rubber bands…everything’s very bureaucratic and very…ehh…I understand that…I understand that they have a job to do and they have their statistics and they have…but it affects people like me who are…who are in a damn poor condition…no, I was in a very poor condition then. And what they do is they squash you even more flat and they tell this man that…you’re worthless because you haven’t progressed farther; you should be here by now…sorry…I’m already lying on my back but you’re welcome to kick me too, that’s alright, go ahead…ehh…that was tough. (Interview 11)

#### Getting mixed messages and sitting in-between

Some participants even experienced that different authorities’ professionals started quarrelling about their work ability and capacity to work during the reconciliation meeting. Having to sit and listen to others discussing their abilities, inabilities and future and arguing about it was described as humiliating and as feeling meaningless.

#### Disrespectful encounters

The participants described being disrespectfully treated at the negative encounters. The disrespect was expressed, for example, in the professionals’ behaviour and language. A participant described a situation with a physiotherapist:

I4: …it was the last time, when I’d been there for a month and I couldn’t…I was supposed to lie on my back and I was supposed to do that…and I can’t lie on my back. I can’t. It won’t work.

L: Mmm, they wanted you to do some training you couldn’t cope with?

I4: Yes. Then she said like: -There’s nothing wrong with you, it’s only in your head. Then I never went back. Of course if you cut my head off I won’t hurt anymore.

L: The physiotherapist said this to you?

I4: Yes she said that. There’s nothing wrong with you, it’s only in your head. (Interview 4)

#### Respectful encounters

The participants described the positive encounters as allowing them to meet with fine professionals who asked what they wanted and how they felt, to be listened to, and to receive helpful rehabilitation. A few participants described entirely positive encounters with professionals, but most of them had both positive and negative experiences.

## Discussion

The most conspicuous findings in the present study were that stopping working brought many changes, that the participants had feelings of powerlessness in the process, and that they experienced offensive treatment and/or encounters with professionals. The uncertainty in waiting while being sick-listed seemed to be especially stressful for the participants. This is earlier described in one study of returning to work [12]. Ockander and Timpka’s [8] findings, that after some time of sick leave the experience turns from positive into a vicious circle of inactivity and isolation, confirm the present study’s findings of a changing process not only in everyday life but also personally. Being away from work changed who they were and this seemed to make them dislike themselves. Previous studies on sickness-absent individuals also describe lowered self-image and self-efficacy [6,7,10-16].

The participants’ sick-listing caused by their illness changed their social status. The social science theory of *status incongruence*, first described by Vernon and Buffler [30] and later by Lundberg, Kristenson and Starrin [31], addresses this problem*.* Being in status incongruence entails a discrepancy in one’s status positions; for instance, having a high education but a low-status job or, as in the present study, not being able to cope with working due to illness and becoming dependent on allowance. This status difference (incongruence) is psychologically stressful, causes shame, and is related to health problems in the long run [30,31]. Thus, due to their illness the participants “lost” their status when they lost control over crucial decisions in their own lives, and as a result felt stressed and ashamed. Some studies suggest that it is the shaming experience that is the depleting negative power in the change processes for both people who are sickness-absent and people with bad finances [32,33].

No longer being able to work and support oneself, and therefore feeling like a “loser”, is also most likely also influenced by norms in society. Swedish people are known to highly appreciate independence, and it is a political and social norm that one should stay independent and contribute to the welfare state by working. In alignment with “the Swedish model” [34], you have the right to receive benefits when your work ability is reduced or disappears altogether due to illness or disability. Still, the norm is to work; thus not working means not contributing, not being “normal”, and being excluded from society. According to the norm in society, the participants in the present study did not fit in and they described the feeling of being stigmatized. Similar experiences of feeling stigmatized are described in, for instance, a study from the United Kingdom in which people with chronic fatigue syndrome experienced claiming welfare benefits as very distressful and as causing feelings of stigma [16].

The overall feeling of losing one’s independence seemed to be closely linked to the fact that the participants were obliged to follow the steps in the rehabilitation chain. If they did not do as the regulations said, they would not get their allowance. They had lost their independence in choosing what would be best to do in order to recover, and they did not have the power to say no. Some of the participants who had had especially bad experiences seemed to have developed a permanent distrust of the professionals they met. The above-mentioned theory of incongruence has been found to be associated with a lack of social trust and to undermine confidence in economic and political institutions [31]. So, the participants’ experiences of status incongruence in combination with disrespectful encounters most likely contributed to the impaired confidence they seem to have in authorities.

### Methodological discussion

The present study presents subjective data, and the researcher is a tool in the analytic process. For trustworthiness, Lincoln and Guba’s qualitative criteria for conducting and assessing qualitative research were followed [35]. Credibility was striven for by attaining the phenomenological attitude through the analysis and the description of the findings. To bracket preconceived assumptions, the first author wrote down her thoughts about the experience of being sick-listed before starting the interviews. Another important step was to avoid reading theory literature that might influence the analysers to discovering themes which “fit” into existing theories or models while forming the phenomenon’s structure. The authors also discussed how their preconceived ideas affected the search for structure in themes and essences throughout the analysis process. The descriptions of the study in the methods section account for the criterion of dependability. When presenting the findings, the main and revelatory themes were consolidated in quotes from the interviews to allow the reader to go back to “the things themselves”, as described by Husserl [24] and as a part of striving for confirmability. The study was discussed with researchers outside the research group several times at seminars to enhance reflection during the analysis and to form confirmability. The sample is small but nevertheless it is most likely similar to long-term sick listed patients in primary health care centres in Sweden, except for the loss of sick-listed with foreign background. Transferability to these settings should be possible. We do not know the reason to why the invited with foreign background declined participation, but several of them had difficulties to communicate in Swedish when contacted by telephone. To offer use of interpreter might have changed their decision. How their experiences would affect the findings is unknown. The present findings can be useful for all professionals working with sick-listed persons in trying to understand how the everyday life of a sick-listed person can change, and how to offer appropriate support. The participants were all ill, and it was not easy to distinguish the experience of sick leave from that of illness. This should be kept in mind when reading this paper.

## Conclusions

Sick-listed can experience the process of being on long-term sickness absent as very negative. The negative experiences are linked to consequences of stopping to work, consequences of social insurance rules and to negative encounters with professionals handling the sickness absence. Experiences can also be positive, but in the present study it was few. Issue for further research are to examine how sick-listed experience participation in the process. There is also a need to further examine how extensive the negative experiences are, and how they affect sick-listed people’s recovery and return to work.

## Competing interests

The authors declare that they have no competing interests.

## Authors’ contributions

LL performed the interviews. All authors participated in the design and critically revised the analysis and the manuscript. All authors read and approved the final manuscript.

## Pre-publication history

The pre-publication history for this paper can be accessed here:

http://www.biomedcentral.com/1471-2458/13/745/prepub
